# Improved Performance of Polysulfone Ultrafiltration Membrane Using TCPP by Post-Modification Method

**DOI:** 10.3390/membranes10040066

**Published:** 2020-04-07

**Authors:** Yuandong Jia, Shuangqing Sun, Shunshun Li, Zhikun Wang, Fushan Wen, Chunling Li, Hideto Matsuyama, Songqing Hu

**Affiliations:** 1 School of Materials Science and Engineering, China University of Petroleum (East China), Qingdao 266580, China; s15090905@s.upc.edu.cn (Y.J.); sunshuangqing@upc.edu.cn (S.S.); z16090707@s.upc.edu.cn (S.L.); wzhikun0806@163.com (Z.W.); fushanwen@upc.edu.cn (F.W.); lichunling@upc.edu.cn (C.L.); 2 Institute of Advanced Materials, China University of Petroleum (East China), Qingdao 266580, China; 3 Center for Membrane and Film Technology, Department of Chemical Science and Engineering, Kobe University, Kobe 657-8501, Japan

**Keywords:** ultrafiltration membrane, porphyrin, protonation, synergistic effect

## Abstract

Ultrafiltration (UF) membranes have found great application in sewage purification and desalination due to their high permeation flux and high rejection rate for contaminants under low-pressure conditions, but the flux and antifouling ability of UF membranes needs to be improved. Tetrakis (4-carboxyphenyl) porphyrin (TCPP) has good hydrophilicity, and it is protonated under strongly acidic conditions and then forms strong hydrogen bonds with N, O and S, so that the TCPP would be well anchored in the membrane. In this work, NaHCO_3_ was used to dissolve TCPP and TMC (trimesoyl chloride) was used to produce a strong acid. Then, TCPP was modified in a membrane with a different rejection rate by a method similar to interfacial polymerization. Performance tests of TCPP/polysulfone (PSf) membranes show that for the membrane with a high BSA (bovine serum albumin) rejection, when the ratio of NaHCO_3_ to TCPP is 16:1 (wt.%), the pure water flux of membrane Z1 16:1 is increased by 34% (from 455 to 614 Lm^−2^h^−1^bar^−1^) while the membrane retention was maintained above 95%. As for the membrane with a low BSA rejection, when the ratio of NaHCO_3_ to TCPP was 32:1, the rejection of membrane B2 32:1 was found to increase from 81% to 96%. Although the flux of membrane B2 32:1 decreased, it remained at 638 Lm^−2^h^−1^bar^−1^, which is comparable to the reported polymer ultrafiltration membrane. The above dual results are thought to be attributed to the synergistic effect of protonated TCPP and NaHCO_3_, where the former increases membrane flux and the latter increases the membrane rejection rate. This work provides a way for the application of porphyrin and porphyrin framework materials in membrane separation.

## 1. Introduction

The increasing industrial and domestic use of water and serious pollution levels have caused a critical shortage of clean water resources [1,2], hence various water purification methods have been developed. Compared with these methods, membrane separation technology has received much attention, mainly due to the fact that it is cost-effective, simple to operate, and eco-friendly [2,3,4]. Among them, the ultrafiltration membrane has attracted significant attention in membrane separation because of the high permeation flux and high rejection rate for these contaminants such as proteins, bacteria, and organic particles under low-pressure conditions [5,6,7,8,9]. In the literature, different polymer materials, such as polyethersulfone (PES) [10,11] and polyvinylidene fluoride (PVDF) [12,13] have been employed as UF membranes. In particular, the polysulfone (PSf) UF membrane has attracted a lot of attention [14]. The PSf family is promising because of their high chemical and thermal stability, which enables a broad operating temperature/pH range and excellent chlorine tolerance. However, one disadvantage of PSf resin is the hydrophobicity, which makes it easy to interact strongly with solutes and hence influence the overall properties of UF membranes, especially water permeability and antifouling ability [15,16,17,18].

As a result, various methods and hydrophilic materials have been utilized to improve the hydrophilicity of PSf UF membranes, such as coating [19,20] and grafting [21,22,23] which can improve both the hydrophilicity and flux of PSf UF membranes. However, the obvious deficiency of such coating is that modifiers may detach from the membrane surface over time [21], and the surface grafting is often limited by a complex synthetic process, and inevitably, grafted polymers cause pore blockage which can reduce membranes’ permeability. Furthermore, the aggressive treatments may cause UF membrane damage [22,24,25]. Another useful method is blending hydrophilic materials to improve UF membrane hydrophilicity. The addition such as polyvinyl pyrrolidone (PVP) [26,27], poly (ethylene glycol) (PEG) [28,29], TiO_2_ [30,31] and ZnO [32] can improve membrane separation performance and antifouling ability, but in the above-mentioned methods, it is always difficult for membranes to maintain a long-term stable separation performance.

Therefore, we need to explore new materials and methods to improve the water flux and antifouling ability of PSf UF membranes. Recently, porphyrins and porphyrin frameworks, as new membrane materials, have attracted more and more attention for the following reasons: (1) the maternal body of porphyrin and porphyrin framework is porphin [33], which is a class of planar conjugated macrocyclic molecular structure, resulting in the porphyrin and porphyrin frameworks materials with good chemical stability and water stability. (2) The position on the peripheral ring of the porphyrin is easily substituted by other organic groups, leading to a widely versatile property for the porphyrin compound and these also will greatly increase the types of porphyrin framework materials and provide a valuable resource for the selection of porphyrin membrane materials. (3) The nitrogen in the porphyrin ring was protonated when encountering strong acid, and then formed a hydrogen bond with the element with a lone pair of electrons, so that the porphyrin could be well anchored in the membrane [34,35].

**Figure 1:**
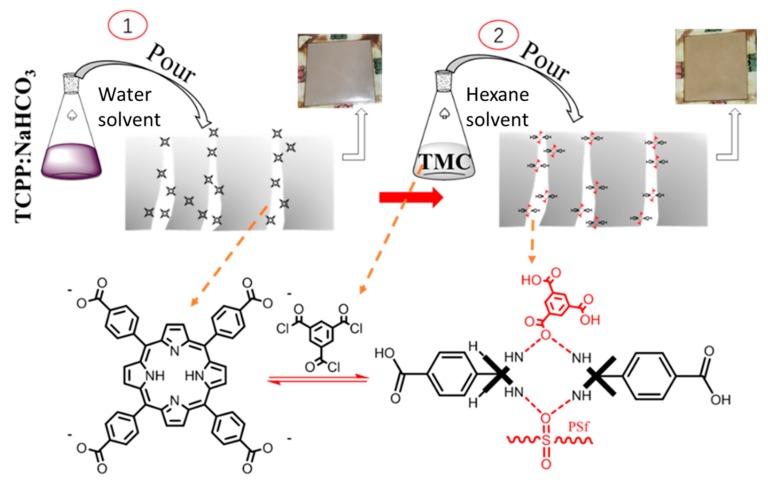
Schematic diagram of tetrakis (4-carboxyphenyl) porphyrin/ polysulfone (TCPP/PSf) membranes synthesis route.

In this work, we used a method similar to interfacial polymerization to directly modify TCPP (tetrakis (4-carboxyphenyl) porphyrin), which has good hydrophilicity and dyeing property into a PSf UF membrane. Since TCPP is hardly soluble in water, it can be dissolved by weakly alkaline NaHCO_3_, mainly to form porphyrin sodium salt. The hydrolysis of TMC (trimesoyl chloride) was first used to produce a transient strong acid to promote the protonation of TCPP, thereby achieving the purpose of fixing TCPP in the membrane (see Figure 1); at the same time, TMC hardly harms the membrane. Membrane separation performance tests indicate that protonated porphyrin membranes can both increase pure flux and increase the rejection rate. Then, analysis showed that the cause of this result was the synergistic effect of protonated TCPP and NaHCO_3_, in which protonated TCPP membranes can increase membrane flux. Furthermore, the NaHCO_3_ can increase membrane rejection rates. The experimental process is simple and convenient to operate and this method offers a potential possibility for applying porphyrin and porphyrin framework membrane materials to water treatment.

## 2. Experimental Method

### 2.1. Materials

Polysulfone base membrane B1 and B2 were supplied by SEAPS Science and Technology Co., Tangshan, China, and the specific parameters are shown in Table 1. The main difference of the above two membranes is that the BSA (bovine serum albumin) rejection of the B1 membrane is 98.67%, and that of the B2 membrane is 81%, corresponding to a low water flux (112.36 Lm^−2^h^−1^bar^−1^) and a high-water flux (677.52 Lm^−2^h^−1^bar^−1^), respectively. Polysulfone (Ultrason S 6010, *Mw* = 60,000 g/mol and glass transition temperature *T_g_* = 187 °C) was supplied by BASF China (Mainland) (Shanghai, China). N-N-dimethylacetamide (DMAc ≥99.5%), and polyethylene glycol (average *Mn* 600) were obtained from Sinopharm (Shanghai, China). 4-formylbenzoic acid (98%), trimesoyl chloride (98%) and pyrrole (99%) were purchased from SIGMA-ALDRICH (Shanghai, China). Propionic acid, sodium hydroxide, hydrogen chloride, tetrahydrofuran, sodium bicarbonate and hexane were obtained from Sinopharm. Bovine serum albumin (BSA, pI = 4.8, Mw = 67,000 g mol^−1^) was obtained from Shanghai LanJi (Shanghai, China). All chemicals were used as received. Deionized (DI) water (18.2 MΩ cm) was used throughout the experiment.

### 2.2. Preparation and Characterization of TCPP (tetrakis (4-carboxyphenyl) porphyrin)

TCPP was synthesized according to the Adler method [36]. First, propionic acid (100 mL) and 4-formylbenzoic acid (6.3 g, 0.042 mol) were mixed in the round bottom flask with the condenser tube attached, and the temperature was raised to 100 °C. After 4-formylbenzoic acid was completely dissolved, the pyrrole (3.0 g, 0.043 mol) was added. The temperature was adjusted to 130 °C, and the reaction was refluxed for 12 h in darkness. The products were vacuum filtered and dried in an oven for 20 h at 40 °C. The dried product was then dissolved in 0.01 mol/L NaOH adjusting the solution pH to 10–11, with the insoluble substances removed by filtration. The pH of the filtrate was adjusted to 3–4 with 0.1 mol/L HCl. Finally, the obtained products by vacuuming filtration were washed with a large amount of deionized water, and dried in vacuum to yield dark purple crystals.

To characterize the as-prepared TCPP, FTIR (Fourier Transform infrared spectroscopy) spectra were obtained via the Bruker Tensor 27 FTIR spectrometer (Bruker, Germany) to analyze the chemical composition, proton nuclear magnetic resonance (^1^H NMR) spectra were obtained via the Bruker AV 400 spectrophotometer (Bruker, Germany) at room temperature using DMSO-d_6_ as the solvent, and the characteristic ultraviolet absorption peak of TCPP was measured by the Hitachi UV-3900 spectrometer (Tokyo, Japan). The yield of as-prepared TCPP was 18%, ^1^H NMR (300 MHz, DMSO-d_6_): δ 8.85 (s, 8H), 8.36 (d, 8H), 8.33 (d, 8H), −2.93 (s, 2H). EI-MS: m/z (C_48_H_30_N_4_O_8_) calculated: 790.79, Measured value: 791.19. UV-Vis (DMF): *λ* nm: 417 (Store band); 514, 548, 589, 645 (Q band).

### 2.3. Preparation of Base Membrane

In this work, the base membranes of UF membranes were also prepared by the phase-inversion method. First, PSf (22 wt.%), PEG-600 (22 wt.%), and DMAc (56 wt.%) were added into the round bottom flask, adjusting the temperature to 90 °C, and the substances were dissolved under constant stirring for about 10 h. Then, the mixed solution was sonicated (80 KHz) for 1.5 h and degassed for 10 h at 25 °C in order to sufficiently remove the bubbles. Next, the mixture was cast onto a glass plate using a glass rod (rounded to 0.15 mm thick copper wires at both ends) and then immersed into a water bath. The water bath was refreshed twice with DI water in the first 30 min, to remove residuals. Then, the membranes were kept in water for at least 12 h. Finally, the membranes were rinsed with DI water and put in a bag with a small amount of DI water, and stored at 4.8 °C. The parameters of self-made base membranes labeled as Z1 are shown in Table 1.

### 2.4. Preparation of TCPP/PSf Ultrafiltration Membrane 

Here, a method that is similar to interfacial polymerization was used to modify the base membrane by the introduction of TCPP (Figure 1). In this method, both aqueous solutions (i.e., TCPP in water and NaHCO_3_) and organic solution (i.e., TMC in n-hexane) were used. The concentration of TMC in n-hexane organic solution was kept at 0.05 wt.%, while different ratios of NaHCO_3_ and TCPP in aqueous solution were configured to achieve optimal performance. The corresponding solution concentration and the membrane labels are shown in Table 2. First, the aqueous solution containing TCPP and NaHCO_3_ was poured on the top surface of the polysulfone base membrane. After soaking for 3 min, the residual solution on the base membrane was rinsed off with 50 mL DI water, and then the water drops on the surfaces of the membrane were carefully removed using filter paper. The n-hexane solution containing TMC was then gently poured onto the surface of the above obtained polysulfone base membrane, and after 3 min of reaction, the polysulfone surface was rinsed with n-hexane. After several seconds, the surface of the membrane became dry and soaked in DI water for more than 6 h. Finally, the membranes were rinsed with DI water and put into a bag which contains a small amount of DI water, and stored at 5 °C.

### 2.5. Characterization of Base Membrane and TCPP/PSf Ultrafiltration Membranes

These membranes, including base membrane and TCPP/PSf ultrafiltration membranes, were dried in a vacuum oven for 24 h before measurement. Element analysis was conducted through an energy-dispersive spectrometer (EDS), which was equipped with a field-emission scanning electron microscope (FE-SEM, Ultra 55, Zeiss, Oberkochen, Germany). X-ray photoelectron spectroscopy (XPS, JPS-9010 MC, JEOL Ltd, Tokyo, Japan) was used to analyze the composition inside and outside surface membranes. Before using XPS for inside membrane testing, the non-woven layer (PET (polyethylene terephthalate)) of the membrane is scraped off with a knife, and then etched at 10 nm. FTIR spectra were obtained to analyze the chemical composition of the membrane surfaces, and the UV spectra were obtained using tetrahydrofuran (THF) to dissolve membranes (Hitachi UV-3900 spectrometer, Tokyo, Japan). The ^1^H NMR spectra of membranes dissolved in DMSO-d_6_ solution were measured by a Bruker AV 400 spectrophotometer at room temperature.

Membrane structures, including surface and cross-section morphologies, were observed by scanning electron microscope (SEM JEOL JSM 7500F, Tokyo, Japan). No additional sample preparation was required for the surface observation, while cross-section samples were prepared by freeze-fracturing the membranes in liquid nitrogen. All samples were coated with gold before observation. Atomic force microscopy (AFM, Bruker MultiMode 8, Bruker, Germany) was used to analyze the roughness of the membrane surface. The average roughness (*Ra*) and the root mean square of the Z data (*RMS*) were collected. Contact angles, tested by DSA 100 (KRUSS, Hamburg, Germany), were used to characterize the hydrophilicity of membranes. Averaged results were obtained by the examination of five different areas of each sample. The thermogravimetric analysis of B2 base and B2 32:1 was conducted under a nitrogen atmosphere with a NETZS 209F3 thermogravimetric analyzer (Frankfurt, Germany) at a heating rate of 8 °C per minute from 30 to 700 °C. For different types of base membranes, the porosity of membranes was measured using the classical gravimetric method [37] which is given by the following:

(1) $$\varepsilon =\frac{W_{wet}-W_{dry}}{AL\rho }\times 100\%$$

where *W_wet_* and *W_dry_* are the weights of the wet and dry membranes, respectively, ρ is the pure water density, *A* is the area of the sample membrane, and *L* is the thickness of the sample membrane. The average pore size was calculated using the experimental permeation and porosity data according to Equation (2):

(2) $$r_{m}=\sqrt{\frac{\left(2.9-1.75\varepsilon \right)\times 8\mu lJ}{\varepsilon \Delta P}}$$

where μ is water viscosity (8.9 × 10^−4^pa s), l is the membrane thickness (m), J is the flux (m^3^h^−1^m^−2^), ΔP is the operational pressure (Pa).

### 2.6. Test of TCPP/PSf Ultrafiltration Membranes Properties

The pure water flux and Bovine serum albumin (BSA) protein rejection of the membrane were measured via a stirred dead-end filtration cell with an effective membrane area of 7.06 cm^2^. In the measurement of pure water flux, the membrane was pre-pressurized at 0.15 MPa for 30 min, then the pressure was reduced to 0.1 MPa for 15 min. The pure water flux ($J_{J1}$) of the membrane was calculated by the following:

(3) $$J_{J1}=\frac{M}{\rho A\Delta t}$$

where *M* (kg) is the mass of water passing through, ρ refers to the density of the permeated water (1.0 g/cm^3^), *A* (m^2^) is the effective area of the membrane (7.06 cm^2^), and Δt (h) is the collecting time of water pass mass. In the measurement of BSA rejection rate, the absorbance of standard BSA solutions with five different concentrations was measured and a standard curve was first obtained. Then, a 1 g/L BSA feed solution was filtrated at 0.1 MPa for 20 min and then the permeated solution was monitored. The rejection rate was calculated according to the following:

(4) $$R=\left(1-\frac{C_{p}}{C_{f}}\right)\times 100\%$$

where *R* is the rejection rate of BSA, $C_{f}$ and $C_{p}$ are the BSA concentration in the feed solution (1 g/L) and the permeated solution, respectively. All parallel samples were tested 3 times.

The antifouling performance of some membranes with large pure water flux (Z1 base, Z1 16:1, B2 base, B2 32:1) were evaluated. The BSA solution (1 g/L, pH = 7) was used to measure the antifouling properties of the membranes, and the process lasted for 60 min. Then, the membrane was washed via DI water for 20 min, and then the pure water flux ($J_{J2}$) was tested lasting for 35 min. The above process was repeated twice. The membrane antifouling ability was evaluated by the flux recovery ratio (*FRR*), the reversible fouling ratio (*R_r_*), the irreversible fouling ratio (*R_ir_*) and the total fouling ratio (*R_t_*). The calculation equations are given as follows:

(5) $$FRR\%=\frac{J_{J2}}{J_{J1}}\times 100\%$$

(6) $$R_{r}\%=\left(\frac{J_{J2}-J_{JR}}{J_{J1}}\right)$$

(7) $$R_{ir}\%=\left(1-\frac{J_{J2}}{J_{J1}}\right)$$

(8) $$R_{t}\%=\left(\frac{J_{J1}-J_{JR}}{J_{J1}}\right)$$

where $J_{JR}$ is the flux of foulants.

## 3. Results and Discussion

### 3.1. Characterization of TCPP and Its Protonation in the Membrane

#### 3.1.1. Characterization of as-Prepared TCPP

**Figure 2:**
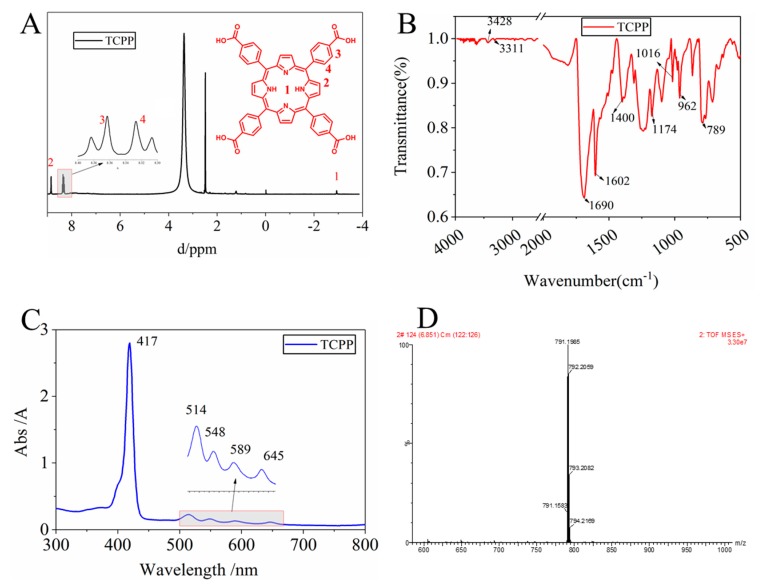
Characterization of as-prepared TCPP, (**A**) ^1^H NMR, (**B**) FTIR spectra, (**C**) UV spectra and (**D**) MS spectra.

In order to determine that the as-prepared product is the target product, the as-prepared product was characterized by ^1^H NMR, FTIR, UV, and MS. The ^1^H NMR spectrum, Figure 2A, clearly shows four hydrogen chemical shift peaks, confirming the presence of TCPP. The carboxyl hydrogen chemical shift cannot be observed mainly due to its high activity. The Q band and the S band of the synthetic substances can be seen in the UV spectra (Figure 2C), indicating the formation of a porphyrin macrocycle in TCPP. In the IR spectra (Figure 2B), a characteristic absorption peak of C–N (3311 cm^−1^, 962 cm^−1^) appears and the measured molecular mass of TCPP is consistent with the calculated value (Figure 2D). These spectra confirm the successful synthesis of the targeted TCPP.

#### 3.1.2. Determination of the Existence and Protonation of TCPP in the TCPP/PSf Membranes

**Figure 3:**
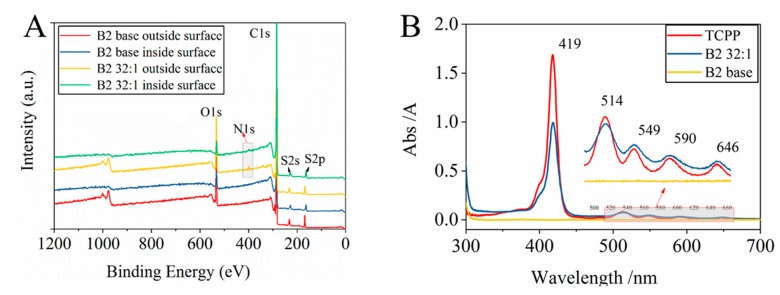
Characterization of TCPP in the membrane. (**A**) XPS spectra of B2 Base and B2 32:1 membrane, (**B**) UV spectra of as-prepared TCPP, B2 Base and B2 32:1 membrane.

After the modification of PSf, in order to characterize the existence of TCPP in the membrane, some membranes with good separation performance were selected for XPS, UV absorption spectroscopy. The XPS spectrum of both B2 32:1 and B2 base membranes are presented in Figure 3A. Several peaks, including O1s, C1s, S2s and S2p appear at the same energy level for both B2 32:1 and B2 base membranes, which confirms that the majority of the membrane is PSf in TCPP/PSf membranes.

In XPS spectra, the peak of N1s appears on both the outside surface and the inside of the B2 32:1 membrane at 398 eV, but not in any position of B2 base membranes (Figure 3A). From Table 3, the content of N element both on the outside membrane surface or inside the membrane increases significantly from 0.96% and 0.89% (in the B2 base membrane) to 1.71% and 1.48% (in the B2 32:1 membrane), which is attributed to the formation of the porphyrin rings containing N element. In UV spectra (Figure 3B), there is no absorption peak seen in the B2 base membrane, but the B2 32:1 membrane shows obvious characteristic absorption peaks of porphyrin, including S band (419 nm) and Q band (514 nm, 549 nm, 590 nm, 646 nm) [38]. These characterizations confirm the existence of porphyrin in the membrane and that TCPP is distributed throughout the membrane.

**Figure 4:**
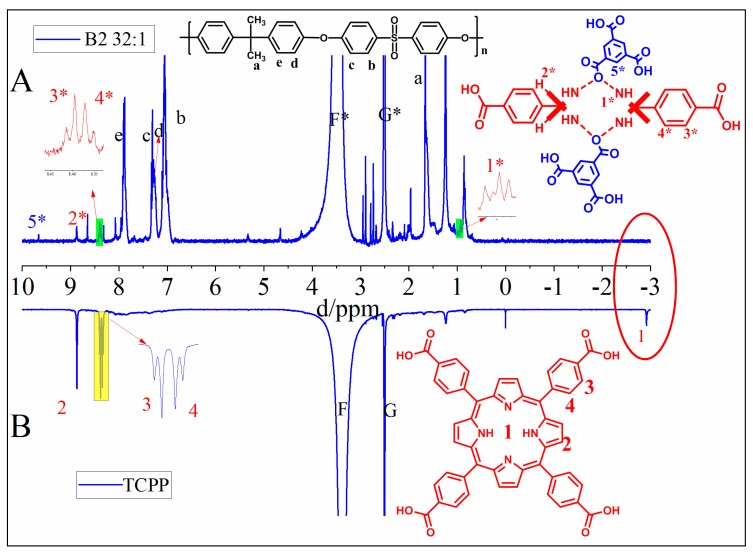
^1^H NMR spectra of (**A**) B2 32:1 membrane and (**B**) as-prepared TCPP.

To confirm the protonation of TCPP, the ^1^H NMR was obtained by dissolving B2 32:1 membrane in DMSO-d_6_ solution (Figure 4A). For comparison, the ^1^H NMR spectrum of as-prepared TCPP was also measured (Figure 4B). From Figure 4A, the hydrogen chemical shift peaks of polysulfone and the hydrolyzed products of TMC are observed, and the hydrogen chemical shift peaks porphyrin ring can be seen such as 2*, 3*, 4*. Looking at the red oval circle in Figure 4, the most notable feature is that the chemical shift peak of hydrogen on the nitrogen in the TCPP ring disappears at about -3 ppm comparing with the ^1^H NMR spectrum of as-prepared TCPP, and a new hydrogen chemical shift peak appears at about 0.9 ppm. This proves that TCPP has been protonated and formed a hydrogen bond with O, S and hydrolyzed acids generated by TMC, causing the hydrogen chemical shift of the TCPP ring to move to a low frequency [34,39].

From UV spectra (Figure 3B), the UV absorption peak of porphyrin S band (419 nm) in membrane B2 32:1 is much weaker than that in as-prepared TCPP, while the absorption peaks of Q band (514 nm, 549 nm, 590 nm, 646 nm) are enhanced. This might be caused by the protonation of TCPP. Besides, the UV absorption change is also consistent with the significant color change of the membrane from brown to yellow when TMC solution was poured onto the membrane surface (see Figure 1).

#### 3.1.3. Distribution and Adhesion of Protonated TCPP in the Membrane

**Figure 5:**
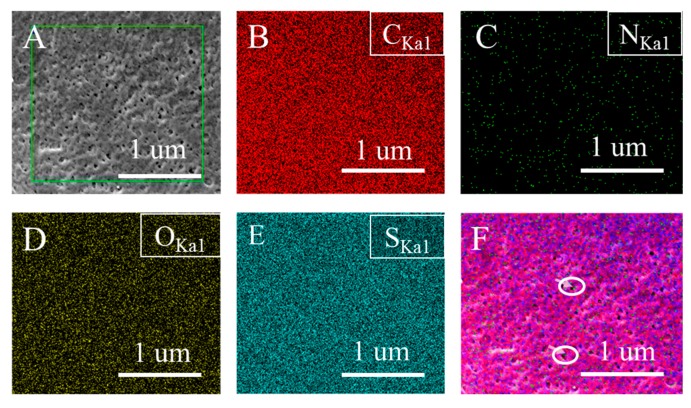
(**A**) The SEM micrograph of the top surface of B2 32:1 membrane and the corresponding elemental mapping analyses of (**B**) Carbon, (**C**) Nitrogen, (**D**) Oxygen, (**E**) Sulfur. (**F**) mixed distribution of all elements (N is in bright green).

The distribution of protonated TCPP in the membrane can be obtained from both XPS and EDS analyses. From Figure 3A and Table 3, it has been found that the percentage of N atoms in B2 32:1 membrane (outside membrane surface) was significantly increased compared with the B2 base membrane (inside membrane). This means that protonated TCPP is distributed throughout the whole thickness of the membrane. The SEM micrograph of the membrane surface and corresponding elemental mapping results are shown in Figure 5. It can be seen that C, O, and S elements are uniformly and continuously distributed on the surface of the B2 32:1 membrane (Figure 5B, D and E). However, in Figure 5C, the N element is sparsely distributed on the surface of the membrane and there is no aggregation phenomenon, indicating that the distribution of N element is relatively uniform and the content is relatively small. In addition, we also found that most of the N elements are distributed in the lower part of the membrane surface or the pores of membrane surface considering their relatively consistent locations between N element and the membrane surface morphology (Figure 5F).

**Figure 6:**
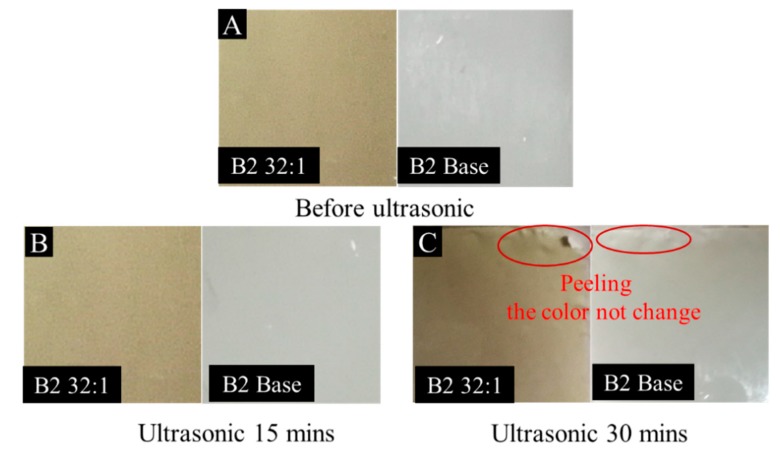
Digital photographs of B2 Base and B2 32:1 membrane before (**A**) and after ultrasound (**B**: ultrasound 15 mins; **C**: ultrasound 30 mins).

In order to determine the adhesion of protonated TCPP in the membrane, B2 32:1 and B2 base membranes were selected for ultrasound at 80 kHz for 30 min. It was found that the color of the protonated porphyrin membrane did not change after 30 min of ultrasound (see Figure 6). At the same time, the membrane layer and the PET non-woven layer began to peel off at the edge of the membrane in Figure 6C. This indicates that the protonated TCPP has good adhesion for PSf membranes. The main reason for this is that protonated TCPP and polysulfone resin form strong hydrogen bonds, which makes TCPP adhere more firmly in the membrane.

### 3.2. Characterization of TCPP/PSf Membranes

#### 3.2.1. Morphology and of TCPP/PSf Membranes

**Figure 7:**
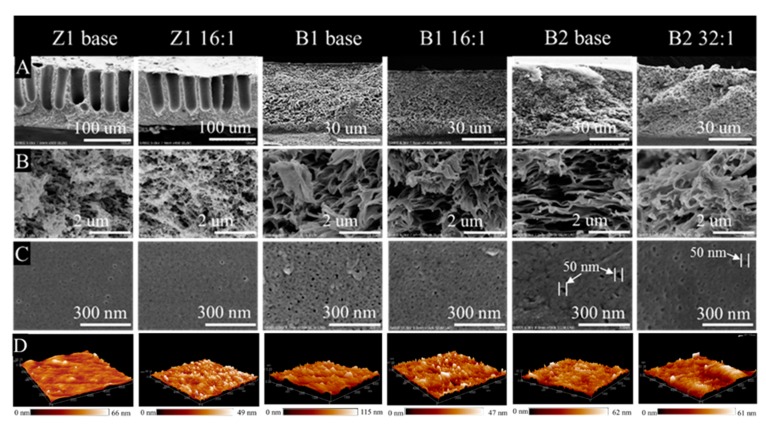
Morphology of membranes. (**A**) and (**B**) cross-sectional SEM images, (**C**) surface SEM images, and (**D**) AFM images of three-dimensional surfaces of the membranes.

The morphological structures of membranes were further observed by SEM and AFM. Figure 7A shows two different cross-sections of membranes, including a large finger-like pore morphology in Z1 base and Z1 16:1 membranes, and a sponge-like pore morphology in B1 base, B1 16:1, B2 base, and B2 32:1 membranes. It also shows that the total thickness of the Z1 membranes is about 100 µm, which is more than twice the thickness of other membranes. From the enlarged cross-sections (Figure 7B), the dense layers of Z1 membranes are also porous and sponge-like, which is similar to that of B1 and B2 membranes. In addition, the dense layer thickness of Z1 membranes is about 25–40 µm, which is comparable with that of B1 and B2 membranes. From enlarged membrane surfaces (Figure 7C), small pores are distributed on the membrane surface. The pore sizes of B1 base, B1 16:1, Z1 16:1 and Z1 base membranes are relatively small and evenly distributed on the membrane surface, and the maximum pore diameter is less than 40 nm. However, large pores are found to irregularly distributed on the surfaces of B2 base and B2 32:1 membranes. Some pores are larger than 40 nm in diameter and the surfaces are uneven. This can lead to a lower rejection rate and higher water flux, which is consistent with the measurements shown in Table 1. The porosity of TCPP/PSf membranes does not show obvious change compared the base membrane (see Figure S1) mainly due to post-modification.

#### 3.2.2. Hydrophilicity and Surface Roughness of TCPP/PSf Membranes

**Figure 8:**
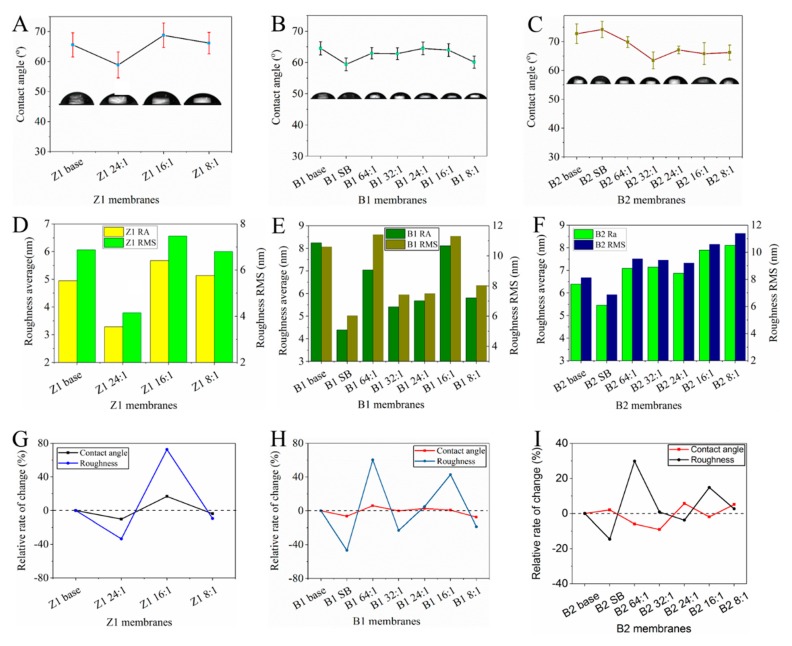
Contact angels of (**A**) Z1, (**B**) B1, and (**C**) B2 membranes, and the surface roughness and root mean square of (**D**) Z1, (**E**) B1, and (**F**) B2 membranes, and relative change rate of contact angle and roughness of (**G**) Z1, (**H**) B1, and (**I**) B2 membranes.

The hydrophilicity of membranes is related to the antifouling performance of the membrane. The contact angle is used to evaluate the membrane hydrophilicity (Figure 8). In general, both the base membranes and the TCPP/PSf membranes are hydrophilic considering contact angles of these membranes are all smaller than 75°. For the base membrane, the Z1 membrane and the B1 membrane (contact angles of 65° and 63°) are more hydrophilic than the B2 membrane (contact angles of 73°). With the increase of the TCPP content in aqueous solution, the hydrophilic angles of the Z1 and B1 membranes undergo little change (Figure 8A,B), while B2 membranes become more hydrophilic compared to their base membrane (Figure 8C).

The roughness of the membrane surface was measured by AFM. AFM images and quantified results are shown in Figure 7D and Figure 8, respectively. Generally, an increase in the surface roughness of the membrane means an increased filtration area of the membrane, which is expected to increase the flux of the membrane [40]. In this work, we can find that for different membranes, the addition of TCPP takes different effects on changing the surface roughness. For the membranes using Z1 base membrane with medium water flux, TCPP does not change the membranes surface roughness very much with the exception of Z1 24:1 membrane (see Figure 8D). For the membranes using B1 base membrane with low water flux, the surface roughness was greatly decreased as the concentration of TCPP increased, with the exception of B1 64:1 and B1 16:1 membranes (see Figure 8E). However, in the case of the membranes using B2 base membrane with high water flux, the surface roughness was slightly increased with the increase of TCPP content (see Figure 8F). Moreover, the roughness of the membrane surface is significantly reduced only when NaHCO_3_ is added or its content is high (such as Z1 24:1, B1 SB and B2 SB).

By comparing the change trend of the contact angle and roughness of the TCPP/PSF membranes, the rate of change of the hydrophilic angle and the roughness was calculated. The changing trends of the roughness and hydrophilic angle of the B1 and Z1 membranes are surprisingly consistent (see Figure 8G,H). The B2 membranes exhibit the opposite changing trend, that is, as the roughness increases, the hydrophilic angle decreases, except for the B2 8:1 membrane (see Figure 8I). These changes suggest that the low rejection rate of B2 membranes and the high rejection rate of B1 and Z1 membranes will appear in different separation performances. 

### 3.3. Properties of TCPP/PSf Membranes

#### 3.3.1. Water flux and BSA rejection of TCPP/PSf Membranes

**Figure 9:**
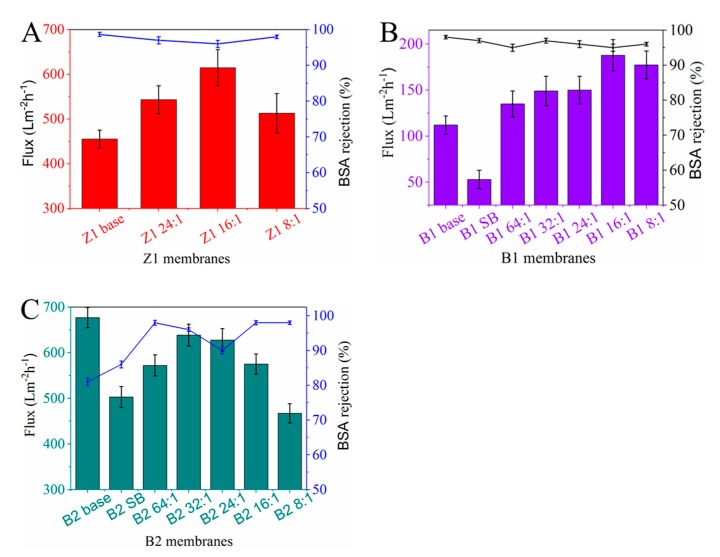
Pure water flux and BSA rejection of (**A**) Z1, (**B**) B1, and (**C**) B2 membranes at a pressure of 0.1 MPa.

The results of pure water flux and BSA rejection of membranes are shown in Figure 9. As shown in Figure 9A,B, the water flux of Z1 and B1 membranes with high BSA rejection (>95%) shows an apparent increase. For the Z1 16:1 membrane, the water flux increases by 34% (from 455 Lm^−2^h^−1^bar^−1^ to 614 Lm^−2^h^−1^bar^−1^). In the case of the B1 16:1 membrane, the water flux even increases by 67% (from 112 Lm^−2^h^−1^bar^−1^ to 187 Lm^−2^h^−1^bar^−1^). Furthermore, in the above two cases, the rejection rates of membranes are almost unchanged compared with their corresponding base membranes. As for B2 membranes with low rejection (Figure 9C), although the flux of TCPP/PSf membranes is slightly decreased, while the rejection rate is obviously increased. In particular, the flux of B2 32:1 stay at a high level of about 638 Lm^−2^h^−1^bar^−1^, and the rejection rate reaches to 96%. This membrane performance mentioned above is found to be comparable to that of some recently reported polymer ultrafiltration membranes at 0.1 MPa [41,42]

As we all know, the membrane permeability is affected by many factors such as membrane morphology, surface roughness, surface pore size, and surface hydrophilicity. In this work, protonated TCPP and NaHCO_3_ are thought to take significant effect to change the membrane properties. Firstly, when only NaHCO_3_ is added (in B1 SB and B2 SB membranes), the roughness of membrane surfaces can be greatly reduced regardless of the different rejection of membranes (see Figure 8E,F), which implies that NaHCO_3_ can reduce the pore size or increase the smoothness of the surface of the membrane. From Figure 9 when only NaHCO_3_ is added, the flux of B1 membranes is significantly reduced, and the rejection of the B2 membrane is significantly increased, which also confirms that NaHCO_3_ can indeed reduce the pore size.

Second, it is known that protonated TCPP itself is hydrophilic. Therefore, when it adheres on pore surfaces in the membrane (see Figure 3A and Figure 5), it can increase the hydrophilicity of the membrane and thereby promotes the increase of water flux. This can explain that as the TCPP content increases, the pure water flux of the membrane increases. However, when the TCPP content is too high (in the case of Z1 8:1, B1 8:1 and B2 8:1 membranes), the water flux starts to decrease. For these trends, the main reason is that some of TCPP aggregated on the membrane surface and adsorbed in the membrane blocks the pores, causing a decrease in water flux and an increase in rejection.

Third, in this work, we find that the optimal separation performance of membranes occurs at different ratios of NaHCO_3_ to TCPP. The synergistic effect from NaHCO_3_ to TCPP can explain why the optimal ratio shifts in different membrane systems. When the base membrane has a high rejection rate, the higher content of dissolved TCPP is beneficial to increase the water flux (Figure 9A,B), considering the hydrophilicity of TCPP. However, when the base membrane has a low rejection rate, a higher content of NaHCO_3_ seems necessary to reduce the pore size of membranes. Therefore, the optimal ratio of NaHCO_3_ to TCPP will shift to a lower TCPP content (Figure 9C) in B1 membranes. In conclusion, the achievement of the optimal separation performance of membranes needs an appropriate ratio of NaHCO_3_ to TCPP. In other words, the usage of TCPP and NaHCO_3_ with proper ratio can take a synergistic effect in improving both membrane flux and rejection of membranes.

#### 3.3.2. Anti-Fouling Performance of TCPP/PSf membranes

**Figure 10:**
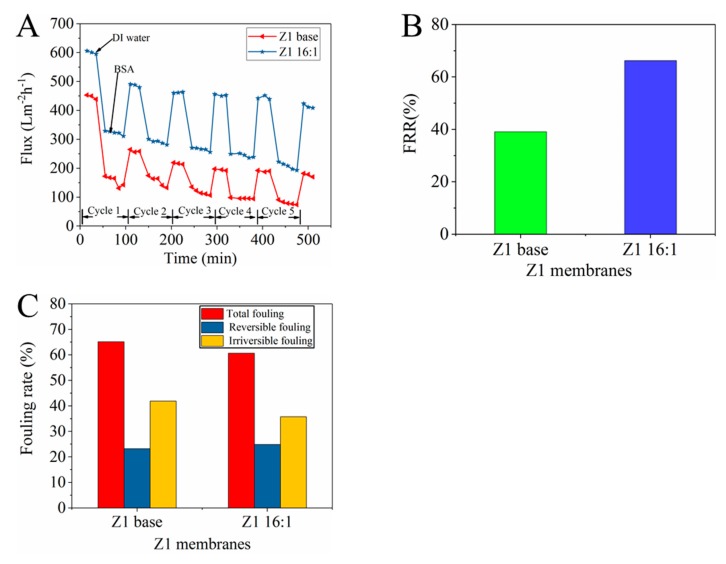
Antifouling properties of the prepared membranes (**A**) time dependent flux, (**B**) flux recovery ratio, and (**C**) fouling resistance.

During the membrane separation process, foulants are easily adsorbed on the membrane surface and block the pores, resulting in severe flux decay [17]. To investigate the antifouling property of membranes, we used BSA as the foulant and performed five cycles of ultrafiltration experiments. From Figure 10A, the water flux of all membranes sharply reduces when the feed solution was changed to the BSA solution. This is mainly attributed to the formation of a layer of protein sediment on the membrane surface. Generally, washing is often employed to eliminate reversible fouling and recover membrane flux.

In this work, fouling recovery rate (FRR) is used to reflect the antifouling performance of the membrane. As seen in Figure 10B, the protonated porphyrin can improve the antifouling performance of the membranes. The FRR of the Z1 membranes is increased from 39% to 66%. The main reason is that the added TCPP can improve the thin-layer hydrophilicity, which thereby increases FRR. The *R_t_*, *R_r_* and *R_ir_* were calculated from pure water flux, fouled solution flux and post-cleaning pure water flux of the membranes. These values were depicted in Figure 10C. The total fouling rate of Z1 base membrane is above 80%, while the *R_t_* of TCPP membrane is much smaller (about 60%), and the reversible fouling of the TCCP membrane is also improved (see Figure 10C). This means that TCPP-modified membranes have good antifouling properties and that the modification does not affect the thermal stability (see Figure S2).

## 4. Conclusions

We successfully synthesized TCPP, and modified it in the membrane for the first time using a method similar to interfacial polymerization. It has also been demonstrated that TMC can protonate TCPP and its hydrolysis product can form hydrogen bonds with protonated TCPP. Then, we tested and characterized the membranes and found that the hydrophilicity, roughness and surface topography of different rejection membranes were not the same. The membrane separation performance test found that the protonated porphyrin membranes can both increase the membrane flux and rejection rate for different rejection rate membranes. These analyses show that the improvement of membranes was attributed to the synergistic interaction of protonated TCPP and NaHCO_3_. Then, the anti-fouling of the membrane was characterized, and the porphyrin membrane was found to improve the anti-fouling of the whole membrane. The synergistic effect between protonated TCPP and NaHCO_3_ opens a way for the application of porphyrin and porphyrin framework materials in the field of membrane separation.

## Figures and Tables

**Table 1:** Performance parameters of self-made and purchased base membranes.

Type	Pure Water Flux (Lm^−2^h^−1^)	BSA Rejection Rate (%)	Total Thickness (μm)	Porosity (%)
B1	112.36 (low)	98.00 (high)	43.80	68.16 ± 3.3
Z1	455.30 (medium)	98.67 (high)	137.60	72.13 ± 4.8
B2	677.52 (high)	81.00 (low)	34.73	77.28 ± 5

**Table 2:** Labels of TCPP/PSf membrane and corresponding aqueous solution concentration.

Labels of TCPP/PSf Membranes	Aqueous Solution Concentration (wt.%)		
	Water	NaHCO_3_	TCPP
Z1 24:1	95.84	4.00	0.17
Z1 16:1	95.75	4.00	0.25
Z1 8:1	95.50	4.00	0.50
B1 SB	96.00	4.00	0.00
B1 64:1	95.94	4.00	0.06
B1 32:1	95.88	4.00	0.13
B1 24:1	95.84	4.00	0.17
B1 16:1	95.75	4.00	0.25
B1 8:1	95.50	4.00	0.50
B2 SB	96.00	4.00	0.00
B2 64:1	95.94	4.00	0.06
B2 32:1	95.88	4.00	0.13
B2 24:1	95.84	4.00	0.17
B2 16:1	95.75	4.00	0.25
B2 8:1	95.50	4.00	0.50

SB is the abbreviation of sodium bicarbonate (NaHCO_3_).

**Table 3:** Elemental percentage of B2 base and B2 32:1 membranes obtained from XPS spectra.

Membrane	Etching Depth (nm)	Atomic Percent (atom %)			
		C	O	N	S
B2 base	0	81.3	14.31	0.96	3.27
B2 base	10	91.5	4.94	0.89	2.46
B2 32:1	0	80.9	11.5	1.71	2.72
B2 32:1	10	92.26	3.65	1.48	2.24

## Supplementary Materials

The following are available online at https://www.mdpi.com/2077-0375/10/4/66/s1, Figure S1: Porosity and mean pore size of (A) Z1, (B) B1, and (C) B2 membranes, Figure S2: TGA curves of B2 Base and B2 32:1 membrane in nitrogen atmosphere.
