# Supplementary material for: Improved Performance of Polysulfone Ultrafiltration Membrane Using TCPP by Post-Modification Method
Source: Membranes (Basel). 2020 Apr 7;10(4):66. doi: 10.3390/membranes10040066 (PMC7231367; doi:10.3390/membranes10040066)
Supplement: Supplementary file 1 [file membranes-10-00066-s001.pdf]

membranes

Supplementary Information

Improved Performance of Polysulfone Ultrafiltration Membrane Using TCPP by Post-Modification Method

Yuandong Jia [1,†], Shuangqing Sun [1,†], Shunshun Li [1], Zhikun Wang [1], Fushan Wen [1,2], Chunling Li [1,2], Hideto Matsuyama [3,*] and Songqing Hu [1,2,*]

[1] School of Materials Science and Engineering, China University of Petroleum (East China), Qingdao 266580, China; s15090905@s.upc.edu.cn (Y.J.); sunshuangqing@upc.edu.cn (S.S.); z16090707@s.upc.edu.cn (S.L.); wzhikun0806@163.com (Z.W.); fushanwen@upc.edu.cn (F.W.); lichunling@upc.edu.cn (C.L.)
[2] Institute of Advanced Materials, China University of Petroleum (East China), Qingdao 266580, China
[3] Center for Membrane and Film Technology, Department of Chemical Science and Engineering, Kobe University, Kobe 657-8501, Japan
† These authors contributed equally to this work.
* Correspondence: songqinghu@upc.edu.cn (S.H.); matuyama@kobe-u.ac.jp (H.M.)

1. Results and Discussions

1.1. Porosity and mean pore size of TCPP/PSf membranes

The porosity and average pore size of these TCPP/PSf membranes were also tested using classical weight loss method (see Figure S1). From Figure S1A, S1B and S1C, it can be seen that the overall porosity of TCPP/PSf membranes is decreasing, as the porphyrin content increases. Although the porosity does not show obvious change compared with the base membranes within a certain margin of error. This is due to the fact that TCPP was introduced after the formation of base membranes in this work, and the protonated TCPP content in TCPP/PSf membranes is very small (section 3.1), which is insufficient to affect the porosity of membranes. In addition, TCPP is a small dye molecule that has little effect on the pore size of the ultrafiltration membrane. Similarly, the addition of TCPP also has little effect on the mean pore size (see Figure 9C).

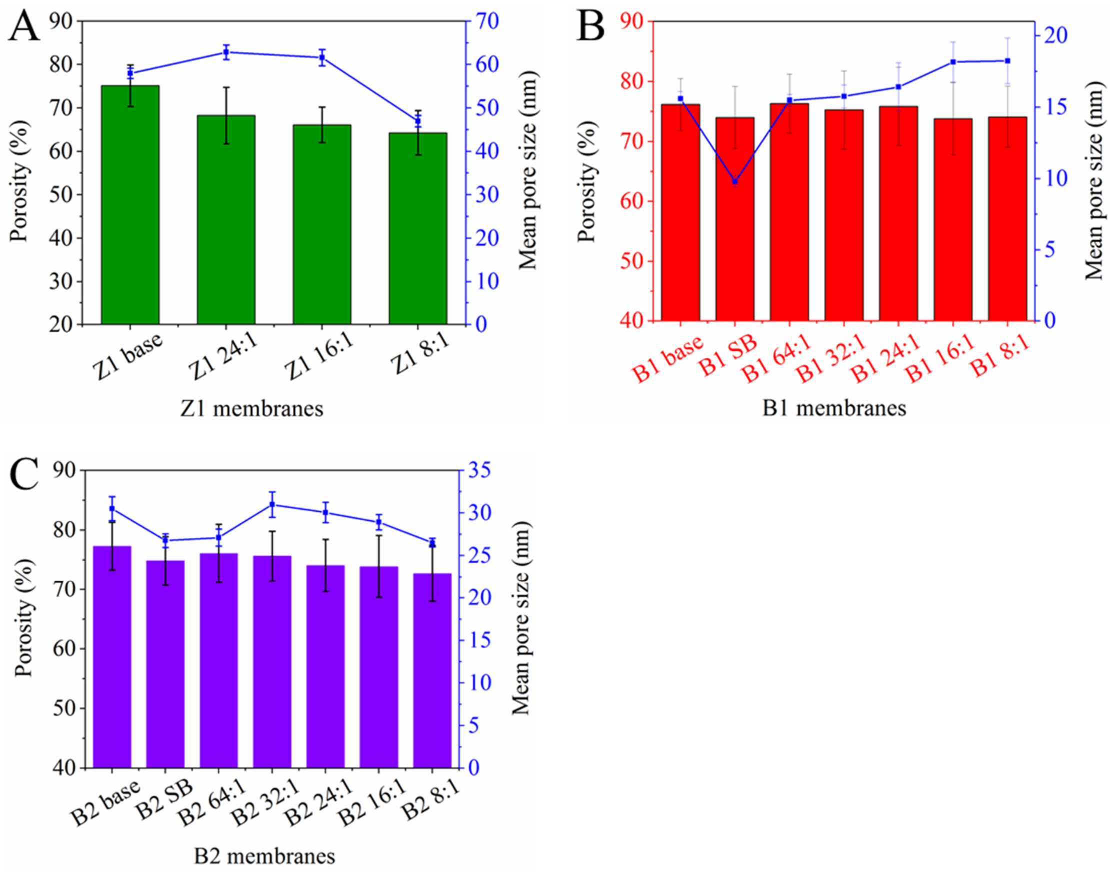

Figure S1. Porosity and mean pore size of (A) Z1, (B) B1, and (C) B2 membranes.

1.2. Thermal stability of TCPP/PSf Membranes

The thermal stabilities of B2 base membrane and B2 32:1 membrane were measured by thermogravimetric analysis in nitrogen atmosphere (Figure S2). Results show both B2 base membrane and B2 32:1 membrane are comparably stable about 450 °C. Difference shows up when B2 32:1 membrane starts to decompose at 480 °C, which is 20 C lower than the decomposition temperature of B2 base membrane. This can be attributed to the decomposition of polysulfone groups[1]. As the temperature further increases, the weight of B2 base membrane decreases faster than that of B2 32:1 membrane. At 550 °C, the weight loss of B2 32:1 membrane is only about 40%, but B2 base membrane has nearly 60% weight loss. This is mainly due to the fact that protonated porphyrin itself is very stable at high temperature, and it reduces the decomposition rate of PSf. But, the thermal stability of protonated porphyrins membranes is much higher than the application requirement of water treatment membranes.

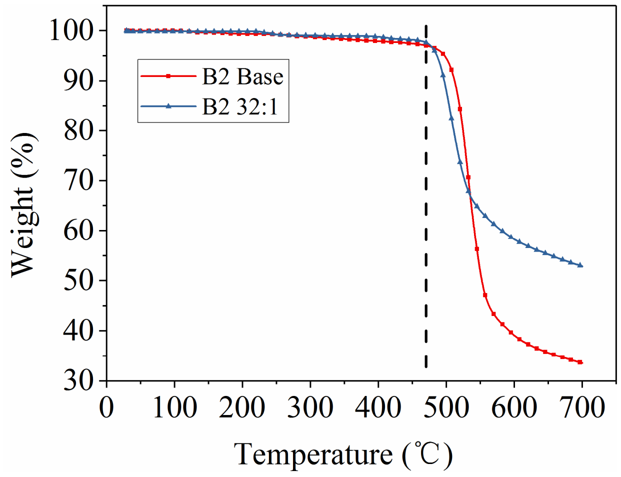

Figure S2. TGA curves of B2 Base and B2 32:1 membrane in nitrogen atmosphere.

References

1. Z. Xu, J. Liao, H. Tang, N. Li, Antifouling polysulfone with pendent sulfonamide groups, *J. Membr. Sci.* **2018**, *548*,481-489.
